# Age-Related Effects on the Color Discrimination Threshold

**DOI:** 10.3390/life15071074

**Published:** 2025-07-05

**Authors:** Ali Almustanyir, Mohammed Alhazmi, Amal Aldarwesh, Meznah S. Almutairi, Mohammed Almahubi, Ansam Alateeq, Tahani Alqahtani, Muteb Alanazi, Sultan Alotaibi, Mansour Alghamdi, Essam Almutleb, Basal H. Altoaimi, Balsam Alabdulkader, Mosaad Alhassan

**Affiliations:** Optometry Department, College of Applied Medical Sciences, King Saud University, Riyadh 11362, Saudi Arabia; malhazmyi@ksu.edu.sa (M.A.); aaldarweesh@ksu.edu.sa (A.A.); mzalmutairi@ksu.edu.sa (M.S.A.); mohammedalmahuby@gmail.com (M.A.); ansamalateeq@outlook.com (A.A.); talqahtani@ksu.edu.sa (T.A.); mkalanazi@ksu.edu.sa (M.A.); salotabi1@ksu.edu.sa (S.A.); algmansour@ksu.edu.sa (M.A.); esalsarhani@ksu.edu.sa (E.A.); baltoaimi@ksu.edu.sa (B.H.A.); alabdulkader@ksu.edu.sa (B.A.); malhassan@ksu.edu.sa (M.A.)

**Keywords:** Konan ColorDx CCT HD, L cone, M cone, S cone, cone contrast sensitivity, wavelength cone systems, color vision

## Abstract

Traditional color vision tests lack the sensitivity to detect subtle differences in individuals with normal color vision. The Konan ColorDx Cone Contrast Threshold (CCT) HD test allows the quantitative measurement of color discrimination thresholds for each cone type. This cross-sectional study established normative values for L-, M-, and S-cone contrast sensitivities and evaluated the effects of age and sex on color discrimination thresholds. Participants aged 15–79 years with normal color vision were included (n = 216; 55% female). CCTs were measured monocularly using the Konan ColorDx CCT HD test under standardized conditions, and the influences of age and sex on L-, M-, and S-cone sensitivities were evaluated. In all groups, L-cone sensitivity was the highest, followed by the M- and S-cone sensitivities. Overall contrast sensitivity was significantly higher in females than in males (mean difference = −0.041), especially for adolescents and young adults (20–24 years). Young adults outperformed middle-aged and older adults, with age-related decline most pronounced for S-cone sensitivity in those over 60. The right and left eye sensitivities did not differ. This study provides age- and sex-stratified normative data for the Konan Color Dx CCT HD test, supporting its use for clinical and occupational assessments.

## 1. Introduction

Before computer-based color vision tests, most clinical tests presented stimuli that were supra-threshold for color vision normal (CVN) individuals. Consequently, these tests could only crudely measure the chromatic thresholds of individuals with color vision deficiencies. The Color Threshold Lantern and Gunkel’s Chromagraph [1,2,3] were exceptions to this, as both could measure color-normal thresholds, but neither has been widely used. In most clinics, the color vision testing protocol starts with a screener test, such as the Ishihara or Hardy–Rand–Rittler (HRR) test. If the patient fails, they undergo the Farnsworth D15 test or continue with the diagnostic series in the HRR test.

Some clinics have replaced printed tests with computer-based ones, such as the Color Assessment and Diagnosis test (COL-Aeglia, Lelystad Airport, NL), Rabin Cone Contrast test (RCCT) (Innova Systems, Burr Ridge, IL, USA), and Cambridge Color Vision Test (Cambridge Research Systems Ltd., Rochester, UK). The Color Assessment and Diagnosis test measures the thresholds for hues that bracket around the lines of confusion of the three types of dichromats; it measures a discrimination ellipse around a gray background [4]. The RCCT estimates the chromatic thresholds for the three cones that might be present in the retina. Although it can estimate the thresholds for color vision defects, it does not present stimuli with sufficiently low chromatic contrasts to measure chromatic thresholds in CVN individuals [5]. The Cambridge Colour Test (CCT) has become one of the most widely used computerized color vision assessments in both clinical and research settings. It comprises two sections: the Trivector test, which measures discrimination thresholds along the protan, deutan, and tritan confusion lines from a gray reference; and the ellipse test, which measures chromatic thresholds for colors equally spaced around a reference color [6]. The CCT’s use of pseudoisochromatic stimuli and adaptive staircase procedures allows for precise quantification of color discrimination thresholds, and it has been validated for detecting both congenital and acquired color vision deficiencies [7,8,9,10]. Numerous studies have used the Cambridge Colour Test to establish normative data across different age groups, to assess the impact of ocular and systemic diseases, and to monitor changes in color vision over time. The number of hues presented, the presence of luminous noise, and the threshold measuring procedures differ between the two tests [4,5]. All three tests have excellent validity for screening red-green color vision defects [7,8,9,10].

**Figure 1:**
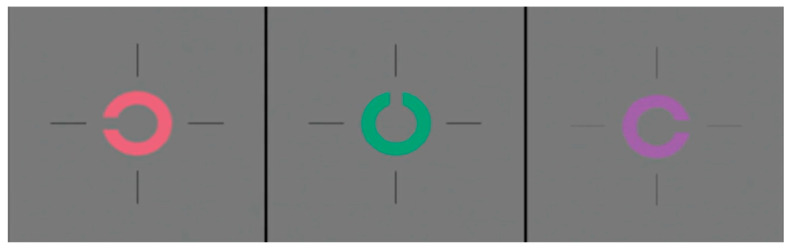
The Landolt C optotype stimulus of L, M, and S cones (red, green, and violet respectively) in the Konan ColorDx Cone Contrast Threshold HD test [11].

Konan Medical and the Occupational Based Vision Assessment Laboratory recently collaborated to produce a commercial version of the Occupational Based Vision Assessment Cone Contrast Threshold (CCT) test, called the ColorDx CCT HD test [11]. The ColorDx CCT HD is a computerized test that provides quantitative threshold information for the three cone types [12]. Furthermore, it utilizes Landolt C with a randomized representation in four directions: up, down, left, and right. Figure 1 shows the test target. In this assessment, the participants are asked to indicate which of the four orientations is presented. The test has separate modes for screening and measuring chromatic thresholds. The screening mode presents each of the three colors corresponding to the individual cone mechanisms at a supra-threshold contrast (i.e., approximately three standard deviations higher than the mean threshold) multiple times. More than one incorrect response for any hue is considered a failure. A prototype evaluation indicated a false-positive screening rate of 37%, possibly because of the strict scoring criterion that only allows one error for each cone mechanism [13]. The commercial version followed the same procedure as that of the prototype. The chromatic threshold measurement mode varies the contrast of the Landolt C by using the Ψ adaptive procedure to determine the individual’s thresholds [14]. The prototype version’s monocular and binocular L-cone and M-cone threshold modes showed excellent agreement with the Rayleigh match in screening for red-green color vision defects [13]. The major advantage of the Konan ColorDx CCT HD test over the RCCT is that it can measure thresholds for CVN individuals, which is not possible with the RCCT since its lowest contrast is fixed and supra-threshold for many CVN individuals.

Despite the broad adoption of the Cambridge Colour Test and other computerized tests, there remains a need for normative data using the Konan ColorDx CCT HD, particularly in color vision normal individuals, to further expand the applicability of computerized color vision testing. This study aimed to establish these values and thus define color thresholds for safely and efficiently performing tasks that require accurate color discrimination.

## 2. Materials and Methods

The ColorDx CCT HD (Konan Medical, Irvine, CA, USA) is a visual function testing device that adaptively evaluates vision by selectively activating the retinal L-cones, M-cones, and S-cones. The test was performed on a desktop computer (all-in-one, Dell Intel CORE i5) with a Windows 7 Professional operating system. The stimuli were presented on a Dell monitor (Model Inspiron 22-3265). The monitor was calibrated using an X-Rite (Version EODIS3 i1) Display Pro colorimeter every 30 days. The luminance of the gray background was 69 cd/m^2^.

The Landolt C projected at the screen center had a size equivalent to 20/317 Snellen acuity, subtending a 1.43° visual angle. Participants viewed a sequence of randomly oriented Landolt-C symbols (with gaps facing up, down, left, or right) against a gray background. Using the keyboard arrow keys, participants indicated perceived gap direction within a 5 s forced-choice window per stimulus, requiring a response regardless of perceptibility. A Bayesian thresholding algorithm dynamically adjusted the LogCS contrast of each subsequent optotype based on the participant’s prior responses. The contrast level of each subsequent stimulus was adjusted upward or downward depending on previous responses (correct or incorrect), using progressively smaller contrast increments to precisely determine the LogCS threshold and reduce the standard error. The test measured the final CCT score thresholds for each cone class, regardless of the screening test results, using the Ψ adaptive threshold. This procedure allowed the measurement of the threshold with a fixed slope of the psychometric function [14]. The company set the slopes to 2.6 for the L- and M-cone tests and 1.9 for the S-cone test. The threshold was determined based on 30 presentations of each cone-isolated stimulus.

The participants viewed the test at 1 m away from the screen, and their task was to press the keyboard corresponding to the direction of the gap in the “C.” The Landolt C was subtended by 1.4°, and the gap was 0.3°. The room was dimly lit (1 lx), and the participants performed the tests monocularly. The eyes tested first were randomized; however, the order of the stimulus presentation was always L-, M-, and then S-cone. The presentation time for each stimulus is around 2 s, and the participant needs 3–4 min to complete the session for each eye.

The King Saud University Office of Research Ethics (E-22-6731) approved this study, which adhered to the tenets of the Declaration of Helsinki. The inclusion Criteria were as follows:

- Adults with no known visual problems other than refractive error.
- Monocular distance visual acuity of 6/9 (corrected or uncorrected) or better in each eye, as measured by the Bailey–Lovie logMAR chart. If presbyopic, participants wore corrective lenses near their current prescription during testing.
- Provided informed consent and completed a screening questionnaire confirming eligibility.

The exclusion Criteria were as follows:

- Any self-reported history of ocular disease, color vision deficiency, or systemic conditions known to affect vision (other than refractive error).
- Use of tinted contact lenses or glasses during testing.
- Failure to meet the visual acuity requirement in either eye.
- Evidence or suspicion of bilateral vision disorders associated with acquired color vision defects.

The color vision test battery included an anomaloscope (Rayleigh equation), Ishihara 38 plates edition (Kanehara Trading Inc., 2011; Tokyo, Japan), HRR plates 4th edition (Richmond Products, Albuquerque, NM, USA), and the Konan ColorDx CCT HD test. The printed tests were illuminated using an LED lamp (NEW POWER, Model no: G45-14 E27, manufactured color rendering index > 90) at a correlated color temperature of 6500 K. The illuminance was set at 400 lx (±5%) in the horizontal plane of a white table where the tests were conducted.

Statistical analysis was conducted using repeated measures ANOVA to evaluate the effects of cone type (L, M, and S), eye (right “OD”, left “OS”), gender (male, female), and age category on cone contrast sensitivity. The eye was treated as a within-subject factor, while age and gender were treated as between-subjects factors. Mauchly’s Test of Sphericity was applied to assess the assumption of sphericity; where sphericity was violated, the Greenhouse–Geisser correction was applied to adjust the degrees of freedom. Type III sums of squares were used for all effects. Between-subjects effects were also analyzed using factorial ANOVA, and pairwise post hoc comparisons were performed using Bonferroni adjustments to control for multiple testing. Effect sizes were reported using partial eta squared (η^2^p), and all analyses were conducted with a significance threshold set at *p* < 0.05. Statistical computations were carried out using SPSS software (Version 29.01, IBM SPSS Statistics Inc., Chicago, IL, USA).

## 3. Results

The analysis included 216 participants (55% female) aged 15–79 years (mean ± standard deviation: 36.3 ± 15.4 years). Age groups were defined according to established health policy frameworks [15] (Table 1).

**Figure 2:**
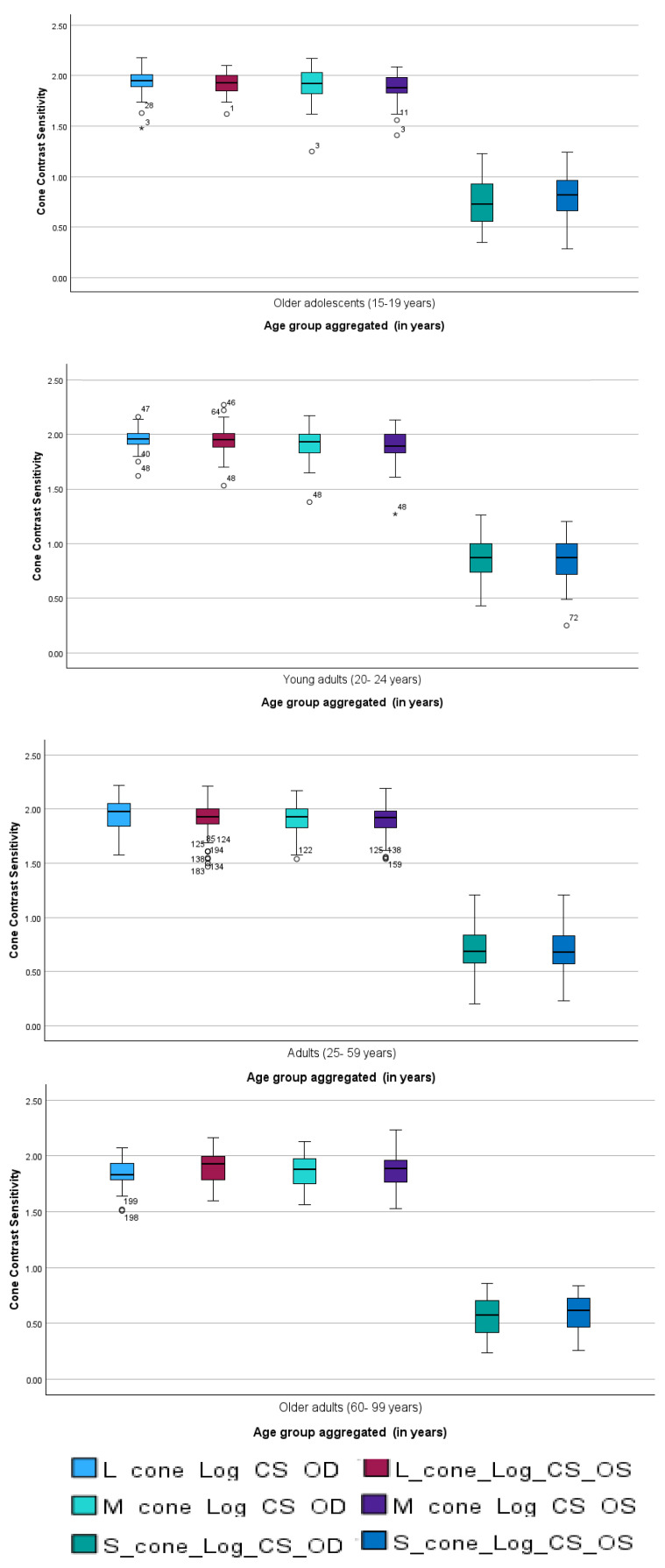
Mean cone contrast sensitivity (L, M, S) across age categories. Age groups follow health policy classifications: 15–19, 20–24, 25–59, and 60–99 years. Values represent group means across both eyes; error bars indicate SD. Circles indicates values that lie beyond the typical range of the data but are not classified as outliers, while asterisk represent statistical outliers identified during analysis. The highest sensitivity was observed in the L-cone, with a general age-related decline evident across all cone types.

To illustrate age-related trends in cone contrast sensitivity, mean sensitivity values for each cone type were plotted across the four age categories (Figure 2). The results demonstrate a gradual decline in contrast sensitivity with increasing age, particularly after early adulthood. Across all age groups, the L-cone consistently exhibited the highest sensitivity, followed by the M- and S-cones.

The right and left eyes demonstrated consistent age-related patterns for the L-, M-, and S-cone contrast sensitivities in both sexes. For male participants (n = 97), the scores across all cone types were higher in the adult group than in the other three groups. For female participants (n = 119), the young adult and older adolescent groups had comparable increases in sensitivity to other age categories. In all groups, the L-cone sensitivity was consistently the highest, followed by the M- and S-cone sensitivities.

**Figure 3:**
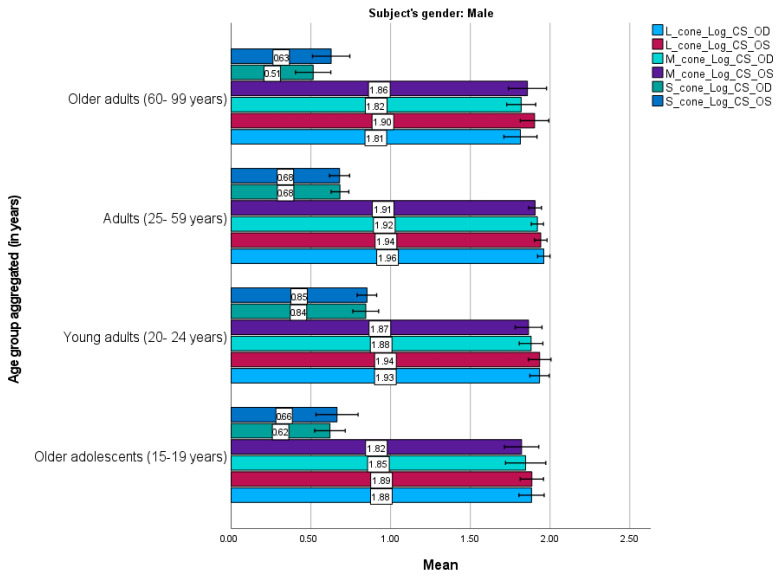
Mean L–, M–, and S–cone scores in male participants (n = 97) based on the Cone Contrast Sensitivity Type and Eye (right eye “OD”, left eye “OS”) tests. Numbers shown on each bar represent the mean value. Error bars represent 95% confidence intervals. OD, OS. Young adults exhibited higher cone sensitivity than older age groups.

**Figure 4:**
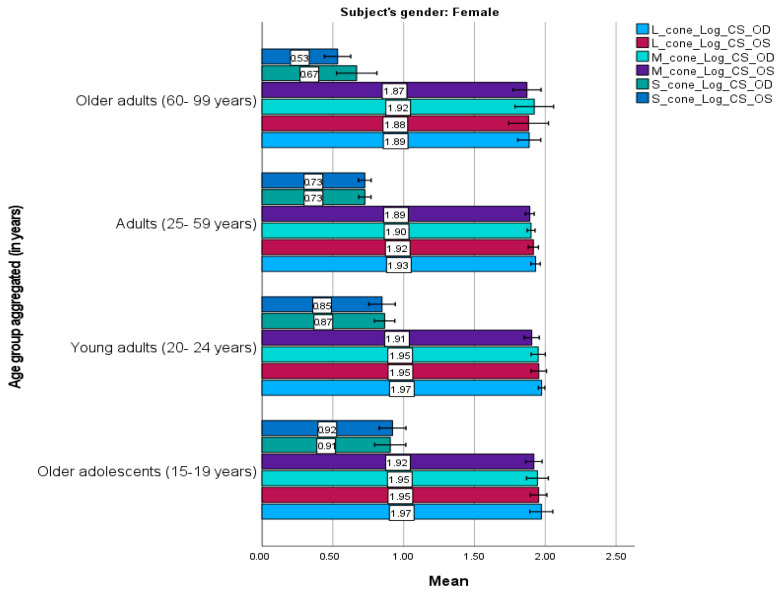
Mean L–, M–, and S–cone scores in female participants (n = 119) based on the Cone Contrast Sensitivity Type and Eye (right eye “OD”, left eye “OS”) tests. Numbers shown on each bar represent the mean value. Error bars represent 95% confidence intervals. OD, OS. Young adults and older adolescents exhibited higher sensitivity than other female age groups.

Figure 3 and Figure 4 present the mean cone contrast sensitivities for males and females, respectively. Similar trends were observed between the male and female participants. They demonstrated similar sensitivities for each cone mechanism for the right and left eyes; the L cone had the highest sensitivity, followed by the M and S cones, consistent with the age-category results. However, slight differences were observed. For instance, adult males had the highest mean sensitivity for all mechanisms, whereas older adolescent and young adult females had the highest sensitivity for the L and M cones, and older adults had the highest sensitivity for the S cone.

Females demonstrated significant improvements in overall contrast sensitivity compared to males (mean difference = −0.041, *t* (208) = −2.535, *p* = 0.012). Age-related analyses revealed that young adults (20–24 years old) outperformed middle-aged adults (25–59 years old; mean difference = 0.050, *t* (208) = 3.067, *p* = 0.015) and older adults (60–99 years old; mean difference = 0.092, *t* (208) = 3.558, *p* = 0.003). Notably, adolescent females (15–19 years) exhibited superior sensitivity to adolescent males (mean difference = −0.118, *t* (208) = −3.429, *p* = 0.020). Cross-sex comparisons further highlighted disparities. For example, young adult females had significantly higher sensitivity than older adult males (mean difference = 0.110, *t* (208) = 3.457, *p* = 0.019).

The sensitivities in the right and left eyes were also compared. In females, the results suggest that the right and left eyes have similar age-related trends, with the highest scores in the L cone, followed by the M and S cones. Additionally, young adult and older adolescent females had similarly higher scores for all cones compared to the other age categories.

Pairwise comparisons confirmed substantial differences between the cone types, with both L-cone (*t* (208) = 71.992, *p* < 0.001, Cohen’s d = 7.354) and M-cone (*t* (208) = 82.672, *p* < 0.001, Cohen’s d = 7.304) sensitivities markedly exceeding that of the S-cone. No significant difference was observed between L- and M-cone sensitivities. These differences remained consistent across age and sex cohorts.

Although sensitivity in the right and left eyes did not differ (F (1, 208) = 1.456, *p* = 0.229), the interactions among contrast sensitivity, sex, and age revealed modest yet statistically significant effects. For instance, the interaction among contrast sensitivity, sex, and age was significant (F (5.480, 379.964) = 3.235, *p* = 0.004) with a small effect size (η^2^p = 0.045). Similarly, the interaction between contrast sensitivity and eye laterality had a small but significant effect (F (1.647, 342.558) = 5.900, *p* = 0.003, η^2^p = 0.028). These findings indicate that demographic variables, particularly age (explaining 7% of variance; η^2^p = 0.070) and sex (3% of variance; η^2^p = 0.030), independently influence contrast sensitivity.

## 4. Discussion

The present study established normative data for L-, M-, and S-cone contrast sensitivities using the Konan ColorDx CCT HD test, finding that age and sex significantly affect color discrimination thresholds in individuals with normal color vision. The L-cone sensitivity was consistently the highest across all age groups and both sexes, followed by the M-cone sensitivity; the S-cone sensitivity was markedly lower. This pattern is consistent with previous physiological and psychophysical data on cone distribution and sensitivity in the retina [16,17]. Specifically, this hierarchy of sensitivity reflects the uneven distribution of cone types in the human retina, where L- and M-cones are far more numerous than S-cones, comprising approximately 60% and 30% of the cone population, respectively, while S-cones represent only about 10%. The predominance of L-cones in the central retina likely contributes to the higher L-cone sensitivity observed in our study. Additionally, the spatial distribution of S-cones, which are largely absent from the central fovea and more sparsely distributed throughout the retina, correlates with their lower sensitivity thresholds in our testing paradigm. This physiological arrangement has been confirmed through adaptive optics imaging, micro-spectrophotometry, and molecular genetic studies of the human retina, all of which demonstrate the numerical and functional dominance of L-cones over M-cones, with S-cones being the least numerous and most vulnerable to aging effects.

We found that age-related decline in color contrast sensitivity was particularly pronounced in older adults (≥60 years), especially in the S-cone pathway, aligning with the established literature. These results indicate that the S-cone system is more vulnerable to age-related changes in the crystalline lens and retinal neural pathways [18,19]. Age-dependent optical changes, such as lens yellowing and reduced pupil size, can disproportionately affect the transmission of short-wavelength light, impairing S-cone stimulation [20].

The observed similarity in cone contrast sensitivity between older adolescents and older adults may reflect a transitional phase in the developmental trajectory of visual function, characterized by a plateau at the end of adolescence and the early onset of age-related decline. This pattern is consistent with prior research indicating that contrast sensitivity reaches its peak in early adulthood and gradually declines thereafter [21,22,23]. Including this observation offers additional context for understanding the continuum of visual sensitivity across age groups.

Neurophysiological studies have also shown that neural transmission in the visual pathways slows with age, affecting contrast sensitivity and chromatic processing [24,25]. The magnocellular and parvocellular pathways, which carry information from the L- and M-cones, may retain their functionality longer than the koniocellular pathway, which is responsible for S-cone signals, which could explain the more pronounced decline in S-cone sensitivity [17,26]. Our current results support these observations, with S-cone thresholds showing the greatest elevation in older adults, suggesting that blue-yellow discrimination is particularly vulnerable to age-related visual decline.

These findings are in line with previous studies using the Cambridge Colour Test, which have also demonstrated age-related increases in chromatic discrimination thresholds, especially along the tritan axis (S-cone pathway) [27]. The CCT has been used to establish normative data for different age groups, and our results with the Konan ColorDx CCT HD are comparable in showing that S-cone sensitivity is most affected by aging. Furthermore, studies utilizing the Cambridge Colour Test have shown its sensitivity to acquired color vision loss in various ocular and neurological diseases (e.g., glaucoma, diabetic retinopathy, multiple sclerosis), supporting the clinical relevance of computerized threshold-based color vision testing [27,28,29,30].

Interestingly, our data revealed a sex effect with females, particularly adolescents and young adults, demonstrating significantly higher contrast sensitivity than males. This result supports those in previous reports suggesting subtle sex-based differences in color perception and contrast sensitivity, potentially due to hormonal influences or X-linked genetic variability affecting cone opsin expression [31,32].

The superior performance of younger individuals across all cone types likely reflects optimal optical clarity, retinal photoreceptor function, and cortical processing efficiency in early adulthood [24]. As individuals age, optical media degrade, and age-related neural losses in the retina and visual cortex may also impair chromatic processing [33].

The high agreement between the monocular and binocular thresholds across the eyes indicates the Konan ColorDx CCT HD test has good test–retest reliability, consistent with earlier validation studies of this platform [11,12]. Our results support its use in occupational settings and clinical monitoring, particularly where subtle color vision changes may affect task performance or indicate early visual dysfunction.

Establishing normative cone contrast thresholds stratified by age and sex has direct implications for clinical diagnostics and occupational vision standards. Color vision assessments have traditionally relied on qualitative screening tools, such as the Ishihara plate, Farnsworth D-15, and HRR tests, which primarily detect congenital red-green color vision deficiencies. However, these tools have limited sensitivity for detecting subtle or acquired dyschromatopsia and do not provide quantitative estimates of chromatic sensitivity [34,35]. In contrast, the Konan ColorDx CCT HD test uses a psychophysically validated adaptive algorithm (the Ψ method) to determine cone-specific contrast thresholds, offering higher resolution and objectivity in clinical assessments [11,12]. The present findings, together with prior studies using the Cambridge Colour Test, underscore the value of computerized, quantitative color vision testing for both research and clinical applications.

In clinical ophthalmology, quantitative CCT can be a functional biomarker for early-stage retinal and optic nerve diseases and has proven useful for detecting early changes in various conditions. For instance, in glaucoma, S-cone sensitivity is often the first factor to affect visual function [36]. Diabetic retinopathy, especially in the presence of subtle macular ischemia or edema, occurs without overt fundus changes [37], and color vision changes may precede visual field defects in optic neuritis and multiple sclerosis [38]. The Cambridge Colour Test has also been employed to detect such early changes in disease, further supporting the utility of computerized color vision assessments.Color sensitivity changes can also indicate drug toxicities, such as early S-cone loss from ethambutol and hydroxychloroquine, and may help guide dose adjustment or discontinuation decisions [39].

In occupational settings, vision requirements vary widely depending on the job demands, and existing screening methods may fail to detect borderline or task-specific deficiencies. For instance, the Federal Aviation Administration and the International Civil Aviation Organization require normal color vision for pilots, air traffic controllers, and aircraft maintenance personnel. Traditional pass/fail tests do not capture the nuanced impairments that could impact safety in these roles [13,40].The Cambridge Colour Test and similar computerized assessments have been proposed as more sensitive alternatives for occupational screening, and the Konan ColorDx CCT HD may offer similar benefits with its rapid, quantitative approach.

This study has several limitations. It is possible that always testing L-, M-, and then S-cones in a fixed order may have contributed to some retinal or perceptual fatigue, especially for the cone type tested last (typically S-cone). This could potentially elevate S-cone thresholds independent of true physiological differences. Randomizing the order of cone testing in future studies would help control for any order effects or fatigue, and clarify whether the observed differences are solely due to cone distribution and aging, or partly influenced by test sequence. It is also important to consider that the wavelength of light associated with each cone type may have influenced the results. L-cones are most sensitive to long wavelengths (red), while S-cones are sensitive to short wavelengths (blue). Short-wavelength light is more susceptible to absorption and scattering by the aging ocular media, which may contribute to the higher S-cone thresholds observed, especially in older adults. Additionally, always testing cones in the L-, M-, and S-cone order could interact with these wavelength effects or introduce order-related biases. Randomizing the order of cone testing in future studies would help clarify whether the observed differences are due to inherent cone physiology, wavelength-specific effects, or procedural factors. Considering both the spectral properties and testing sequence will be important for future work to ensure a robust and unbiased assessment of cone function across the lifespan. Lastly, the sample size was relatively robust and stratified across age and sex; longitudinal data could provide better insights into individual aging trajectories. Additionally, although our exclusion criteria helped control ocular diseases, future studies should examine how systemic conditions, such as diabetes or glaucoma, further modulate cone-specific thresholds.

## 5. Conclusions

This study established age- and sex-specific normative data for L-, M-, and S-cone contrast sensitivities using the Konan ColorDx CCT HD test in individuals with normal color vision. The findings confirm that color discrimination, particularly for the S-cone, declines with age. Additionally, females, especially adolescents and young adults, tend to have slightly better color contrast sensitivity than males. These results highlight the importance of considering both age and sex when interpreting color vision tests. The normative values generated here provide a valuable reference for clinical assessments and setting occupational standards where accurate color perception is essential. The Konan Color Dx CCT HD test is a reliable tool for detecting subtle changes in color vision, which supports its use in clinical and occupational settings. Future research should further validate these norms in broader populations and explore their application in detecting early acquired color vision deficiencies.

## Figures and Tables

**Table 1:** Participant demographics.

Age Category	Sex		
	Male	Female	Total
Older adolescents (15–19 years)	14 (48.3%)	15 (51.7%)	29 (100%)
Young adults (20–24 years)	20 (44.4%)	25 (55.6%)	45 (100%)
Adults (25–59 years)	50 (41%)	72 (59%)	122 (100%)
Older adults (60–99 years)	13 (65%)	7 (35%)	20 (100%)
Total	97 (44.9%)	119 (55.1%)	216 (100%)

## Data Availability

All relevant data are presented within the article.

## Abbreviations

The following abbreviations are used in this manuscript:

CCT	Cone Contrast Threshold
CVN	Color vision normal
HRR	Hardy Rand Rittler
RCCT	Rabin Cone Contrast test
