# Correction: Almustanyir et al. Age-Related Effects on the Color Discrimination Threshold. *Life* 2025, *15*, 1074

**DOI:** 10.3390/life15111771

**Published:** 2025-11-19

**Authors:** Ali Almustanyir, Mohammed Alhazmi, Amal Aldarwesh, Meznah S. Almutairi, Mohammed Almahubi, Ansam Alateeq, Tahani Alqahtani, Muteb Alanazi, Sultan Alotaibi, Mansour Alghamdi, Essam Almutleb, Basal H. Altoaimi, Balsam Alabdulkader, Mosaad Alhassan

**Affiliations:** Optometry Department, College of Applied Medical Sciences, King Saud University, Riyadh 11362, Saudi Arabia; malhazmyi@ksu.edu.sa (M.A.); aaldarweesh@ksu.edu.sa (A.A.); mzalmutairi@ksu.edu.sa (M.S.A.); mohammedalmahuby@gmail.com (M.A.); ansamalateeq@outlook.com (A.A.); talqahtani@ksu.edu.sa (T.A.); mkalanazi@ksu.edu.sa (M.A.); salotabi1@ksu.edu.sa (S.A.); esalsarhani@ksu.edu.sa (E.A.); baltoaimi@ksu.edu.sa (B.H.A.); alabdulkader@ksu.edu.sa (B.A.);

## Affiliation Removal

In the published publication [1], there was an error regarding the affiliation for Sultan Alotaibi. In addition to affiliation 1, the updated affiliation should remove: 2. The authors state that the scientific conclusions are unaffected. This correction was approved by the Academic Editor. The original publication has also been updated.
